# Optimal composite scores for longitudinal clinical trials under the linear mixed effects model

**DOI:** 10.1002/pst.1701

**Published:** 2015-07-30

**Authors:** M. Colin Ard, Nandini Raghavan, Steven D. Edland

**Affiliations:** ^1^ Department of NeurosciencesUniversity of California, San DiegoLa JollaCAUSA; ^2^ Janssen Research & Development, LLCRaritanNJUSA; ^3^ Division of Biostatistics, Department of Family and Preventive MedicineUniversity of CaliforniaSan Diego, La JollaCAUSA

**Keywords:** linear mixed effects model, Alzheimer's disease, mild cognitive impairment, composite, longitudinal clinical trial

## Abstract

Clinical trials of chronic, progressive conditions use rate of change on continuous measures as the primary outcome measure, with slowing of progression on the measure as evidence of clinical efficacy. For clinical trials with a single prespecified primary endpoint, it is important to choose an endpoint with the best signal‐to‐noise properties to optimize statistical power to detect a treatment effect. Composite endpoints composed of a linear weighted average of candidate outcome measures have also been proposed. Composites constructed as simple sums or averages of component tests, as well as composites constructed using weights derived from more sophisticated approaches, can be suboptimal, in some cases performing worse than individual outcome measures. We extend recent research on the construction of efficient linearly weighted composites by establishing the often overlooked connection between trial design and composite performance under linear mixed effects model assumptions and derive a formula for calculating composites that are optimal for longitudinal clinical trials of known, arbitrary design. Using data from a completed trial, we provide example calculations showing that the optimally weighted linear combination of scales can improve the efficiency of trials by almost 20% compared with the most efficient of the individual component scales. Additional simulations and analytical results demonstrate the potential losses in efficiency that can result from alternative published approaches to composite construction and explore the impact of weight estimation on composite performance. Copyright © 2016. The Authors. Pharmaceutical Statistics Published by John Wiley & Sons Ltd.

## 1 Introduction

Recent years have seen a shift in drug development and clinical trial design strategies in Alzheimer's disease (AD), with an increasing emphasis on evaluation of treatment efficacy early in the disease. This evolution, largely motivated from an expectation that earlier intervention will prove critical, has introduced new challenges for trial design because the standard instruments used to quantify cognitive and functional decline in AD are relatively insensitive to the changes that are typical of early stages of the disease. As a consequence, achieving acceptable levels of statistical power with reasonable and obtainable sample sizes has become increasingly difficult. Several strategies for addressing this issue have been considered and evaluated in the literature, including the use of rescoring algorithms 1, and the specification of biomarkers as endpoints 2.

Another approach to improving the efficiency of clinical trials is the use of so‐called *composite* outcome measures composed of a linear combination of the most sensitive items or instruments available for a given condition. For example, regulators from the Food and Drug Administration have released a draft guidance 3 and published a *New England Journal of Medicine* editorial 4, describing the rationale for composite endpoints for preclinical AD. A number of composite endpoints formed by combining scales or subscales from existing clinical, functional, and neuropsychometric assessment batteries have been proposed as primary endpoints for clinical trials of mild cognitive impairment (MCI) and preclinical AD 5, 6, 7. Discarding items or calculating simple sums or averages of component test scores may be inefficient, and a weighted composite that optimizes signal to noise of the constructed outcome would be preferred. To this end, Xiong *et al*. 8 have proposed a weighting algorithm for combining scores from multiple instruments or markers into a single composite endpoint assuming a multivariate linear mixed effects model and a primary analysis plan comparing mean rate of decline in treatment versus control. Their weights outperformed alternative weighting schemes in some reported simulations but were suboptimal in others, leading the authors to conclude that additional research is needed. We show that the suboptimality noted by Xiong *et al*. results from the fact that their proposed weights do not account for the design and duration of the study in which the composite is to be employed. We derive a formula for calculating linear weights from pilot data that accommodate future planned trial design; the weights are optimal under the multivariate linear mixed effects model and can be estimated from pilot study data of arbitrary design provided the number of measurement occasions in the pilot study is sufficient for model identification (in the absence of residual autocorrelation, three visits are sufficient).

Section 2 sets notation through an exposition of model assumptions and briefly reviews the analytic results presented in Xiong *et al*. 8. We then propose optimality criteria and derive a formula, given in Equation (2), for the optimal linear weights as a function of the planned trial design. In Section 3, we estimate weights and demonstrate composite performance using data from the completed Alzheimer's Disease Cooperative Study (ADCS) MCI/donepezil trial 9. Section 4 presents theoretically derived illustrations of composite performance and describes a simulation study conducted to (1) empirically validate the optimality of the proposed weights, and (2) characterize the sampling distributions of weights estimated from pilot data and explore their impact on composite performance. Finally, Section 5 concludes with a discussion of assumptions and other issues relating to the implementation of weighted composites.

## 2 Optimal Weights

### 2.1 Model specifications

Let *y*_*ij*_(*t*) denote the score for subject *i* on outcome measure *j* = 1,…,*m* at time *t*. We assume throughout that the response vector $y_{i}(t)={\left[y_{\mathrm{ij}}(t)\right]}_{j=1}^{m}$ follows a multivariate linear mixed effects model:

$$y_{i}(t)=\alpha +a_{i}+(\beta +b_{i})t+e_{i}(t).$$

Here, $\alpha ={\left[\alpha_{j}\right]}_{j=1}^{m}$ and $\beta ={\left[\beta_{j}\right]}_{j=1}^{m}\neq 0$ are vectors containing the fixed intercept and slope coefficients pertaining to the *m* tests. The corresponding random effects vectors $a_{i}={\left[a_{\mathrm{ij}}\right]}_{j=1}^{m}$ and $b_{i}={\left[b_{\mathrm{ij}}\right]}_{j=1}^{m}$ are assumed jointly normally distributed with *E*{***a***_*i*_}=*E*{***b***_*i*_}=**0**, and arbitrary symmetric positive definite covariance matrix. In what follows, we will make use of the *m* × *m* covariance matrix of the random slopes, which we denote **Σ**_*b*_. Neither the covariance matrix of the random intercepts nor the matrix containing the covariances of the random intercepts and random slopes will be required, so we forego the introduction of notation for these parameters. The vector of residual error terms $e_{i}(t)={\left[e_{\mathrm{ij}}(t)\right]}_{j=1}^{m}$ is also assumed to follow a multivariate normal distribution, independent of the random effects, with *E*{***e***_*i*_(*t*)}=**0**, and *C* *o* *v*{***e***_*i*_(*t*)}=**Σ**_*e*_ for fixed *t*, with **Σ**_*e*_ positive definite but not necessarily diagonal.

Six months is a typical testing interval for clinical trials in AD and MCI. We conjecture that under these circumstances, the impact of residual autocorrelation on the results is likely to be negligible, and restrict attention to the case where no autocorrelation obtains in the examples and simulations to follow. The model above can nonetheless be elaborated as follows to accommodate situations in which serial autocorrelation in the composite residual errors cannot be ignored. Let *γ*_*e*_:[0,*∞*)→[0,1] be an autocorrelation function for which the following two conditions hold:

$$\mathrm{Cov}\{e_{\mathrm{ij}}(s),e_{ij^{\prime }}(t)\}=\gamma_{e}(|t-s|)\cdot e_{j}^{T}\Sigma_{e}e_{j^{\prime }},\;\forall \;j,j^{\prime },s,t,$$

$$x^{T}\Gamma_{e}(t)x>0\;\forall \;x\neq 0,$$

where **e**_*j*_ denotes a conformable vector with a one in the *j*th entry and zeros elsewhere, $t={\left[t_{k}\right]}_{k=1}^{p},(t_{k+1}>t_{k}$ is the vector representation of a set of *p*≥2 fixed measurement times, and **Γ**_*e*_(***t***) is the *p* × *p* matrix with *k*,*k*′ entry $\gamma_{e}(|t_{k}-t_{k^{\prime }}|)$. The first condition requires that *γ*_*e*_(0) = 1, limits the number of parameters in the doubly multivariate model for the *y*_*ij*_(*t*), and in so doing implies that the derived composite will have the same autocorrelation function as the component tests. The second condition thus ensures that the residual autocorrelation matrix for the composite in a trial with design ***t*** will be positive definite.

### 2.2 Composite determination

We wish to determine weights, $w={\left[w_{j}\right]}_{j=1}^{m}\neq 0$, with which to construct a linearly weighted composite *z*_*i*_(*t*;***w***) = ***w*** ^*T*^ ***y***_*i*_(*t*) to satisfy some given optimality criterion. Treating ***w*** as fixed, as when the weights are estimated from pilot data and specified in advance of the trial in which they are to be implemented, this construction implies that *z*_*i*_(*t*;***w***) will itself follow a linear mixed effects model:

$$z_{i}(t;w)=\mu (w)+m_{i}(w)+(\delta (w)+d_{i}(w))t+r_{i}(t;w),$$

where *μ*(***w***) = ***w*** ^*T*^ ***α*** and *δ*(***w***) = ***w*** ^*T*^ ***β*** represent the fixed intercept and slope coefficients, respectively, *m*_*i*_(***w***) = ***w*** ^*T*^ ***a***_*i*_ and *d*_*i*_(***w***) = ***w*** ^*T*^ ***b***_*i*_ are their bivariate normally distributed random counterparts, with *E*{*m*_*i*_(***w***)}=*E*{*d*_*i*_(***w***)}=0, and *r*_*i*_(*t*;***w***) is the residual error, independent of the random effects and normally distributed with *E*{*r*_*i*_(*t*;***w***)}=0∀*t*. As with the individual markers, we require the variance of the random slopes, $\sigma_{d}^{2}(w)=w^{T}\Sigma_{b}w$, the variance of the residual errors, $\sigma_{r}^{2}(w)=w^{T}\Sigma_{e}w$, and the covariances among the residual errors over the set of measurement times ***t***, $\Sigma_{r}(t;w)=\sigma_{r}^{2}(w)\Gamma_{e}(t$.

The *linear mixed effects* (LME) weights $w_{{}_{\mathrm{LME}}}$ proposed later in Equation (2) satisfy both finite sample and asymptotically motivated formulations of optimality. Details are given in Appendix A. For the time being, we motivate the derivation of the LME weights through a brief outline of the asymptotic optimality criterion. Let ${\hat{\delta }}_{n}(t;w)$ denote a consistent, efficient, asymptotically normal estimator – for example, by (restricted) maximum likelihood – of *δ*(***w***) based on a sample of *n* individuals observed at measurement times ***t***. For such an estimator, as the sample size *n* goes to infinity, $\sqrt{n}\left({\hat{\delta }}_{n}(t;w)-\delta (w)\right)\overset{ℒ}{\underset{}{\to }}N\left(0,\sigma_{\hat{\delta }}^{2}(t;w)\right)$, where $\sigma_{\hat{\delta }}^{2}(t;w)$ is the diagonal entry corresponding to *δ*(***w***) of the inverse of the Fisher information matrix for a single *p*‐variate observation from the composite longitudinal distribution (Appendix A). With standard hypothesis testing and interval estimation procedures – for example, evaluating *H*_0_:*δ*(***w***) = 0 against *H*_1_:*δ*(***w***) ≠ 0, or *H*_2_:*δ*(***w***) > 0 when *δ*(***w***) is in fact positive – the limiting probability of detecting a nonzero slope in the expected, or true, direction on the composite is increasing in $\left|\delta (w)|/\sigma_{\hat{\delta }}(t;w\right)$. This leads to the following criterion for determining an optimal weight vector ***w*** ^∗^:

(1) $$w^{*}\in {\text{argmax}}_{w\neq 0}\;\left\{\frac{\delta {\left(w\right)}^{2}}{\sigma_{\hat{\delta }}^{2}(t;w)}\right\}.$$

As we show in Appendix A, this condition is satisfied by choosing $w^{*}=w_{{}_{\mathrm{LME}}}(t)$, as given by

(2) $$\begin{array}{cc} w_{{}_{\mathrm{LME}}}(t) & =c{\left[\Sigma_{b}+\tau_{e}(t)\Sigma_{e}\right]}^{-1}\beta \\ =c\Lambda_{t}^{-1}\beta . \end{array}$$

Here, *c* ≠ 0 is an arbitrary constant, and *τ*_*e*_(***t***) is a scalar design term defined as

$$\tau_{e}(t)=e_{2}^{T}{\left[X^{T}\Gamma_{e}^{-1}X\right]}^{-1}e_{2},$$

where **X** = [**1**,**t**], with **1** a conformable vector of 1s. In the special case where there is no residual autocorrelation, **Γ**_*e*_(***t***) = **I**, and $\tau_{e}(t)={\left[\overset{p}{\underset{k=1}{\sum }}{\left(t_{k}-\bar{t}\right)}^{2}\right]}^{-1}$, with $\bar{t}=p^{-1}\overset{p}{\underset{k=1}{\sum }}t_{k}$. This parallels a basic result in the single‐variable LME model with no residual autocorrelation, for which the contribution of the residual variance to uncertainty in the slope estimate is minimized, and hence, precision in the slope estimate is maximized, by maximizing the sum of squares of the measurement times 2. Note that by previously stated assumptions, all the required inverses exist, and **Λ_***t***_** is positive definite.

The choice of the constant *c* in Equation (2) does not have any impact on composite efficiency. Nonetheless, some additional comment on the role that it plays is warranted. In addition to determining the scale of the composite, *c* is also the sole determinant of the sign of the composite slope, such that $E\left\{z_{i}\left(t;w_{{}_{\mathrm{LME}}}(t)\right)\right\}$ is increasing (decreasing) in *t* whenever *c* is greater (less) than zero, as follows immediately from the fact that $\delta \left(w_{{}_{\mathrm{LME}}}(t)\right)$ is a *c* multiple of a quadratic form in a positive definite matrix. This is, in particular, regardless of the componentwise sign of ***β***. While this might at first seem to suggest, for example, that when *c* > 0 and *β*_*j*_>0 the weight assigned to the *j*th marker will always be non‐negative, this is not necessarily the case, as we show by example in Appendix A. Informally, these ‘negative weights’, when they occur, indicate that the efficiency of the composite is better served by noise cancellation than by signal augmentation. The practical and conceptual challenges posed by negative weights are considered in Section 5. Weight estimation and its consequences for trial efficiency are considered in Sections 4 and 5. A simulated data set and R 10 code that can be used to generate fully worked examples of the LME weighting approach are available for download from the journal website.

### 2.3 LME weights for between‐group comparisons

The presentation above presumes that the composite is being optimized for analysis of rate of change in a one‐group setting, and it is therefore reasonable to ask whether the weights given in Equation (2) are appropriate for trial designs that feature placebo and treatment arms. In general, when treatment affects expectations but not covariances, the optimal weights for detection of treatment‐mediated between‐group differences in rate of change can be determined as follows:

(3) $$w_{{}_{\mathrm{LM}E_{2}}}(t)=c\Lambda_{t}^{-1}\left(\beta_{\mathrm{Trt}}-\beta \right),$$

where ***β***_*Trt*_ is the mean rate of change for the treatment group and ***β*** is the mean rate of change for the placebo group. The derivation of Equation (3) parallels that of Equation (2) after replacing *δ*(***w***)^2^ in Equation (1) with [*δ*_*Trt*_(***w***) − *δ*(***w***)]^2^, say. One obvious challenge in using Equation (3) to determine composite weights from pilot data is the requirement that the relative magnitude of the expected treatment effect on each test be specified in advance of the trial. This difficulty is conveniently bypassed when the treatment effect size, expressed as a proportion of placebo group mean rate of change, is the same for all component tests, that is, when ***β***_*Trt*_=(1 − *k*)***β*** for some nonzero constant *k*≤1, say. In this case, which we refer to as *proportional invariance* (PI), Equation (2) will continue to yield an optimal composite for detection of a 100 × *k* *%* attention of placebo slope owing to treatment. The issue of weight specification for between‐group comparisons is considered in some additional detail in Section 5. In what follows, the term LME weights will continue to refer to the weights of Equation (2) unless otherwise specified.

### 2.4 A related formulation of optimality

The weighting proposal outlined in this paper is in several respects similar to a weighting approach recently described in Xiong *et al*. 8. Although assumptions on covariances differ somewhat from those outlined in the current manuscript, in that Xiong *et al*. assume that residual errors will be independent across tests and allow for distinct residual autocovariance functions from one test to the next, the principal difference between their weights and the LME weights of Equation (2) lies in the optimality criterion each has been derived to satisfy. Under their chosen form of the multivariate LME model, Xiong *et al*. derive their weights to maximize the probability of observing decline in a randomly sampled subject over a one‐unit interval of time, on account of which we will refer to their weights as *unit‐time decline* (UTD) weights, denoted as $w_{{}_{\mathrm{UTD}}}$. As detailed in Appendices A and B, modulo the different assumptions on the covariances among the residual errors and the arbitrary restriction on the direction of change in the resulting composite, UTD weights are in fact a special case of LME weights, where the optimization has been undertaken with respect to a trial with design vector ***t*** = [0,1]^*T*^, instead of with respect to a trial of known, arbitrary design. This difference can have important consequences for composite efficiency, as the simulations of Section 4 demonstrate.

## 3 Example: Lme Weight Calculation

**Figure 1:**
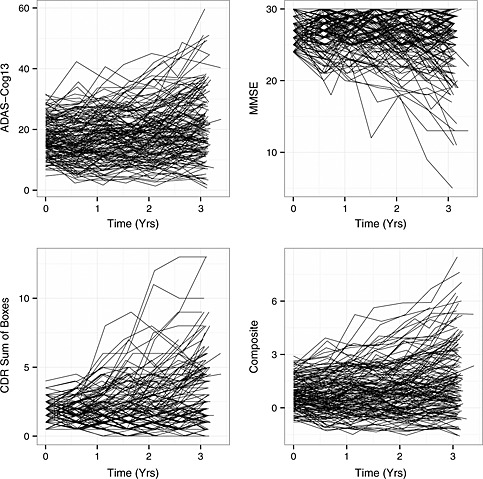
Subject‐specific longitudinal trajectories on the ADAS, CDR, MMSE, and LME‐weighted composite (optimized for 3‐year change from baseline), for *n* = 160 non‐converting completers from the vitamin E arm of the ADCS MCI/donepezil trial 9. Horizontal axes, time on trial (years); vertical axes, test scores.

To illustrate the efficiency gains that are possible with LME‐weighted composites, as well as the manner in which LME weights respond to changes in trial design, we report calculations based on an analysis of data on the cognitive subtest of the Alzheimer's Disease Assessment Scale (ADAS), the Clinical Dementia Rating Scale (CDR)‐Sum of Boxes, and the Mini‐Mental State Examination (MMSE), using biannual testing data from the *n* = 257 subjects in the vitamin E arm of the ADCS MCI/donepezil trial 9 (all analyses conducted using R statistical software 10). The primary endpoint for this trial was time to conversion to AD, and the design incorporated a provision for transitioning subjects who converted to active treatment with donepezil. We therefore excluded post‐conversion/donepezil treatment visits from all analyses. Parameters were estimated by fitting a multivariate LME model with correlated residuals across tests and within measurement occasions using the R function growth() from the lavaan package^11^, version 0.5‐17 (Supporting information). To support interpretation of the weights, scores were rescaled prior to the analysis by dividing each test score by its respective baseline standard deviation (6.05, 0.78, and 1.90 for the ADAS, CDR, and MMSE, respectively). The fit of this model was found to be superior to a model assuming unstructured multivariate normality by both AIC (9081 for LME vs 9092 for unstructured) and BIC (9198 for LME vs 9986 for unstructured). Plots of individual patient trajectories for the *n* = 160 vitamin E arm subjects who completed the trial without converting are presented in Figure 1 (constructed using the R package ggplot2 12). Unstandardized scores on each of the three component tests, as well as for the LME‐weighted composite, optimized for an analysis of change from baseline to last observation over a 3‐year trial, are depicted. The plots were interpreted as providing graphical support for the appropriateness of the assumption of patient‐specific longitudinal trajectories. The similarity of the composite to the ADAS reflects the fact that the (standardized) ADAS received the largest weight of the three component tests with the specified 3‐year trial duration (Table 1). Estimates of the relevant parameters were as follows, with rows and columns ordered ADAS, CDR, and MMSE:

**Table 1:** Weights and sample size reductions for analyses of change from baseline to last observation.

Trial duration (months)	*w*_*ADAS*_	*w*_*CDR*_	*w*_*MMSE*_	*%* *N*‐*reduction*
6	0.37	0.51*	−0.11	18.0%
12	0.44	0.45*	−0.11	17.1%
18	0.53	0.36*	−0.11	17.9%
24	0.61	0.28*	−0.11	19.8%
30	0.69*	0.20	−0.11	15.9%
36	0.75*	0.14	−0.11	9.5%

*w*_*j*_ references the LME weight assigned to test *j* after rescaling each test by its baseline standard deviation and normalizing the weights to sum to 1 in absolute value (ignoring rounding error); *%* *N*‐*reduction* gives the approximate percent reduction in required sample size by the LME‐weighted composite relative to the best performing individual test treating the estimates as the true parameter values; * indicates the most sensitive individual test for each trial duration.

LME, linear mixed effects; ADAS, Alzheimer's Disease Assessment Scale; CDR, Clinical Dementia Rating Scale; MMSE, Mini‐Mental State Examination.

$$\begin{array}{cc} \hat{\beta } & =\left(\begin{array}{l} 0.29\\ 0.74\\ -0.32 \end{array}\right),\;{\hat{\Sigma }}_{b}=\left[\begin{array}{lll} 0.10 & 0.28 & -0.11\\ 0.28 & 1.04 & -0.38\\ -0.11 & -0.38 & 0.17 \end{array}\right],\\ {\hat{\Sigma }}_{e} & =\left[\begin{array}{lll} 0.24 & 0.05 & -0.06\\ 0.05 & 0.51 & -0.07\\ -0.06 & -0.07 & 0.63 \end{array}\right], \end{array}$$

Table 1 presents the resulting LME weights, scaled so that the composite slope is positive and the sum of the absolute values of the weights is 1 (by choosing $c=1/(\underset{j}{\sum }|\Lambda_{t}^{-1}\beta |)$; reported values subject to rounding error), for hypothetical trials ranging from 6 to 36 months in duration with trial endpoints specified as change from baseline to last observation. As the results from Table 1 show, the LME weights prescribe that the CDR should make the strongest contribution to the composite for shorter trials. As the trial duration increases, however, the LME weights deemphasize the CDR, and the ADAS takes over as the dominant component. The asterisks (*) in Table 1 identify the most efficient of the three tests at each trial duration. The CDR is estimated to be the most efficient instrument for trials with a duration of 2 years or less, whereas the ADAS is estimated to be more efficient for longer trials, roughly tracking the patterns in the weights across the spectrum of hypothetical designs. The negative weights for the MMSE reflect the fact that whereas the ADAS and CDR increase over time in MCI patients, the MMSE decreases over time in the same population.

The theoretical improvement in efficiency available by using the weighted composite relative to the best of the three individual tests for each trial design, operationalized as the approximate percent reduction in required sample size for a specified type I error rate and statistical power 2, and calculated assuming the estimates are the true values of the population parameters, is given in the last column of Table 1. Values range from 9.5% to 19.8% and exceed 15.0% for all but the 36‐month trial duration. The impact of weight estimation on expected composite efficiency is considered in the simulations of Section 4. We emphasize that the analysis presented earlier is intended only to be illustrative. For example, although beyond the scope of the current manuscript, the aforementioned adaptive reassignment of subjects who converted to the donepezil arm would warrant more in‐depth consideration in actual practice.

## 4 Composite Efficiency and Weight Estimation

In this section, we consider the efficiency of weighted composites as a function of the weights utilized in their calculation with two primary goals: (1) to illustrate the losses in trial efficiency that can result when suboptimal weighting schemes are employed and (2) to evaluate the performance of composites calculated using LME weights estimated from pilot data. To facilitate the presentation, attention is restricted to the case in which two tests, termed *B* *e* *s* *t* and *W* *o* *r* *s* *t* and following a bivariate LME model without residual autocorrelation, are being considered for inclusion in a composite for a 3‐year trial with annual observations. Parameters of primary interest are

$$\beta =\left(\begin{array}{l} 1\\ 1 \end{array}\right),\;\Sigma_{b}=\left[\begin{array}{ll} 0.5 & \rho_{\mathrm{Slp}}\\ \rho_{\mathrm{Slp}} & 2.0 \end{array}\right],\;\Sigma_{e}=\left[\begin{array}{ll} 2.0 & 0.0\\ 0.0 & 0.5 \end{array}\right],$$

where the tests are ordered *B* *e* *s* *t*, *W* *o* *r* *s* *t*, and *ρ*_*Slp*_∈{0.2,0.5,0.8}. With these specifications, *B* *e* *s* *t* is a relatively efficient test for the hypothetical 3‐year design, and *W* *o* *r* *s* *t* is relatively inefficient. Although they do not influence composite performance when the data are balanced, the remaining parameters were specified as follows: ***α*** = **0**, *C* *o* *v*{***a***_*i*_}=**Σ**_*b*_, *C* *o* *r*{*a*_*ij*_,*b*_*ij*_}=0.5, and $\mathrm{Cor}\{a_{\mathrm{ij}},b_{ij^{\prime }}\}=\sqrt{0.5\rho_{\mathrm{Slp}}}$, *j* ≠ *j*′∈{*B* *e* *s* *t*,*W* *o* *r* *s* *t*}.

### 4.1 Composite efficiency and the choice of weights

**Figure 2:**
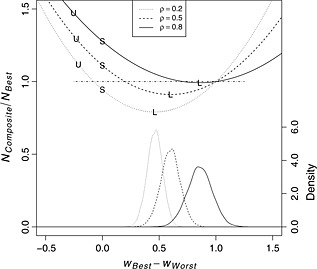
Horizontal axis: *w*_*Best*_−*w*_*Worst*_, difference between weight assigned to the two tests, scaled to sum to 1; left vertical axis: *N*_*Composite*_/*N*_*Best*_, approximate ratio of required sample sizes to detect a nonzero slope in the composite relative to *Best*, with values <1 (dotted‐dashed horizontal line) indicating an efficient composite; right vertical axis: density, kernel density estimate for weight differences based on simulations; *ρ*_*Slp*_, correlation of random slope coefficients between tests; U‐shaped curves in the upper portion of the figure plot *N*_*Composite*_/*N*_*Best*_ as a function of *w*_*Best*_−*w*_*Worst*_ for each value of *ρ*_*Slp*_; points labeled ‘U’, ‘S’, and ‘L’ plot *N*_*Composite*_/*N*_*Best*_ across simulations for UTD‐weighted, inverse baseline standard deviation‐weighted, and LME‐weighted composites; bell‐shaped curves at the bottom of the figure depict kernel density estimates for the *w*_*Best*_−*w*_*Worst*_ values associated with LME weights estimated from simulated data for each value of *ρ*_*Slp*_.

The relationship between the theoretical efficiency of a hypothetical weighted composite and the weight vector ***w*** = (*w*_*Best*_,*w*_*Worst*_)^*T*^ used in its calculation is depicted in Figure 2. Efficiency, on the left vertical axis, is operationalized as $N_{\mathrm{Composite}}/N_{\mathrm{Best}}=[\sigma_{\hat{\delta }}^{2}(t;w)/\delta^{2}(w)]/[\sigma_{{\hat{\beta }}_{\mathrm{Best}}}^{2}/\beta_{\mathrm{Best}}^{2}]$ for a given weight vector ***w***, where $\sigma_{{\hat{\beta }}_{\mathrm{Best}}}^{2}=\sigma_{\hat{\delta }}^{2}(t;e_{1})$. The notation *N*_*Composite*_/*N*_*Best*_ references the fact that the parameter ratio by which it is defined is approximately equal to the ratio of the required sample sizes to detect nonzero slopes with given power in LME model analyses of the composite and of *Best* 2. Values of *N*_*Composite*_/*N*_*Best*_<1 indicate a composite that affords a gain in sensitivity to longitudinal change relative to *B* *e* *s* *t* for the 3‐year design, and values >1 indicate an inefficient composite. A dotted‐dashed horizontal line segment at *N*_*Composite*_/*N*_*Best*_=1 is provided as a visual aid. Weight vectors are registered to the horizontal axis as the difference *w*_*Best*_−*w*_*Worst*_ between the weights under the constraint that *w*_*Best*_+*w*_*Worst*_=1. Thus, for example, a value of 0 on the horizontal axis corresponds to an equally weighted composite, and a value of 1 corresponds to a composite that is identically equal to *B* *e* *s* *t*.

The U‐shaped curves in the top half of Figure 2 plot the relative efficiencies of hypothetical composites as functions of the weights used in their calculation. The minima of the curves indicate the efficiency gains that can be achieved with an optimal linearly weighted composite. For example, for *ρ*_*Slp*_=0.2, the figure indicates that inference based on the maximally efficient composite would require approximately 21*%* fewer subjects than inference based on *B* *e* *s* *t*, whereas for *ρ*_*Slp*_=0.8, the vast majority of potential composites would be less efficient than *B* *e* *s* *t*. The relative ineffectiveness of the compositing approach when *ρ*_*Slp*_=0.8 results in large part from a confluence of two factors: (1) the pronounced difference in the efficiencies of *B* *e* *s* *t* and *W* *o* *r* *s* *t* for the specified design, which limits the value added by incorporation of *W* *o* *r* *s* *t* into the composite regardless of the value of *ρ*_*Slp*_, and (2) the redundancy of the information provided by scores on the two tests when *ρ*_*Slp*_ is large in absolute value.

The points labeled ‘L’ on the three curves indicate the weights and theoretical relative efficiencies of composites calculated using LME weights. Similarly, points labeled ‘U’ refer to UTD weights with the signs reversed and a unit of time defined as 1 year (*cf.*, Xiong *et al*. 8), and points labeled ‘S’ refer to weights that are proportional to the inverses of the baseline standard deviations (*cf.* Donahue *et al*. 6), which are equal for the two component tests in this example. LME weights are located at the minima of the relative efficiency curves, and (asymptotically) optimize power for a composite analysis under the indicated design. By comparison, UTD weights and inverse baseline standard deviation weights are relatively distant from these minimizing LME weights. Expected performance levels of the resulting composites suffer considerably as a result, with *N*_*Composite*_/*N*_*Best*_>1 in five of the six cases depicted for these alternative weighting algorithms. For *ρ*_*Slp*_=0.8, the corresponding efficiency loss approaches a 50% increase in required sample size for the UTD‐weighted composite relative to simply using *B* *e* *s* *t*.

### 4.2 Simulation: estimation of weights from prior data

Parameters required to calculate LME weights are in practice unknown and must be estimated from prior data (although see Xiong *et al*. 8 for a different suggestion). To explore the impact of weight estimation on composite performance, we simulated 10,000 data sets of size *N*_*Train*_∈{100,200,400} using the parameters specified earlier, and for each of the three values of *ρ*_*Slp*_. Parameters for LME weight calculation were estimated from each simulated data set using the summary measures approach outlined in the Supporting information. Primary interest centered on the sampling distributions of the weight estimates as a function of the simulation parameters and *N*_*Train*_, and the expected efficiencies of composites calculated from estimated LME weights.

Kernel density estimates for the estimated weight vectors based on training data sets of size *N*_*Train*_=400 are plotted at the bottom of Figure 2 for each of the three simulated values of *ρ*_*Slp*_ and are registered to the right vertical axis. While some heterogeneity is apparent, the distributions of the differences in the estimated weights are in all three cases approximately symmetrical, centered near the true value of *w*_*Best*_−*w*_*Worst*_ for the LME weights, and reasonably concentrated near the optimizing arguments of the analytic efficiency curves. A more complete cataloging of the findings is provided in Table 2, which gives *N*_*LME*_/*N*_*Best*_ at each value of *ρ*_*Slp*_ assuming $w_{{}_{\mathrm{LME}}}(t)$ known, and also reports the estimated expectations (Expected) and 0.95 quantiles (*Q*_0.95_) of these ratios of estimated sample size requirements from the Monte Carlo simulations at all three values of *N*_*Train*_. As is evident from the table, the expected loss of efficiency from having to estimate the LME weights is quite small (<2%), even with training samples of only 100 subjects. Somewhat greater risk is evident in the estimated 0.95 quantiles, but this also decreases to a practically ignorable level for sample sizes of *N*_*Train*_=400. The amount of precision in weight estimation that is required in any given setting may depend on any number of factors, and the simulations reported here do not obviate the need for sensitivity analyses in the construction of LME‐weighted composites. At a minimum, however, the results reported in Table 2 indicate, in our view, that the need to estimate LME weights should not generally be seen as a barrier to implementation.

**Table 2:** Efficiencies of composites constructed from estimated weights.

*ρ*_*Slp*_	*N*_*LME*_/*N*_*Best*_	*N*_*Train*_=100		*N*_*Train*_=200		*N*_*Train*_=400	
		Expected	*Q*_0.95_	Expected	*Q*_0.95_	Expected	*Q*_0.95_
0.2	0.791	0.805	0.845	0.797	0.817	0.794	0.804
0.5	0.911	0.929	0.979	0.920	0.945	0.915	0.928
0.8	0.992	1.012	1.068	1.002	1.030	0.997	1.011

‘Expected’ gives simulated approximation to $E\{N_{\hat{\mathrm{LME}}}/N_{\mathrm{Best}}\}$, where $N_{\hat{\mathrm{LME}}}$ references the required sample size to detect a nonzero slope in a composite calculated from estimated LME weights, and the expectation is taken with respect to the distribution of the LME weight estimates; *Q*_0.95_ gives simulated approximation to the 95th percentile of the distribution of $N_{\hat{\mathrm{LME}}}/N_{\mathrm{Best}}$.

*N*_*LME*_/*N*_*Best*_, approximate ratio of sample sizes required to detect nonzero slope in LME‐weighted composite relative to *B* *e* *s* *t*; LME, linear mixed effects; *N*_*Train*_, size of simulated pilot data set on which weight estimates were based.

*ρ*_*Slp*_= correlation between random slopes for *B* *e* *s* *t* and *W* *o* *r* *s* *t*.

## 5 Discussion

As demonstrated in the preceding sections, although composite outcome measures offer the possibility of increased efficiency relative to individual tests, improved performance is not guaranteed, and poorly constructed composite instruments can actually decrease statistical power. By contrast, when assumptions are met, LME weights can be used to produce composites with optimal performance characteristics for longitudinal trials. These optimal composites can in some cases dramatically outperform composites calculated using alternative published methods applied to the same pool of assessments. Furthermore, as indicated by the simulations, even when weights must be estimated from pilot data, expected efficiency loss relative to use of the true theoretical LME weights will be of an ignorable magnitude provided pilot sample sizes are sufficiently large. This does not necessarily mean that there is always a great deal to be gained from use of weighted composites. As seen in Sections 3 and 4, in some circumstances, even optimally weighted composites provide minimal performance gains relative to the most efficient of the component tests. Whether or not a composite endpoint should be utilized in such cases is likely to depend on a range of factors, including pilot sample size, regulatory requirements, and the cost of administering multiple tests. The occurrence of ‘negative’ weights, as when the weights for two tests that exhibit change in the same direction take opposite signs, is another situation where exclusion of component tests from the chosen composite may be appropriate. At the very least, in such cases, the potential gains in efficiency from including negatively weighted tests will need to be weighed against the cost to interpretability of the composite.

The choice of the multivariate LME modeling framework in this manuscript owes to several factors. Xiong *et al*. 8 assume a variant of this model in deriving their UTD weights. Further, precedent comes from the fact that the LME model is frequently assumed in studies of AD and MCI 2. Focusing directly on the merits of the LME model with respect to the current application, we perceive two primary strengths. First, the LME model offers a parsimonious yet flexible framework for modeling the changes in means, increases in variance, and decreases in correlations that are typically observed over time in studies of patients with chronic progressive diseases such as AD or MCI. This is likely to be of particular benefit with pilot data sets that feature a relatively small sample size, as the reduction in the number of parameters that must be estimated can be substantial. Second, because LME weights can be calculated for known, arbitrary clinical trial designs given sufficient pilot data, the approach can not only be used to determine weights for trials with a longer duration than available pilot data but can also be adapted for the case where the trial endpoint is change from baseline to last visit – as in a mixed model repeated measures analysis 13 – by specifying the trial design vector **t** = [*t*_1_,*t*_*p*_]^*T*^ (Section 3). In so doing, the primary analysis for the planned trial can be freed from dependence on the linearity assumption, even as the multivariate LME model structure is utilized to reduce the mean square error of the estimates of ***β*** and **Λ_*t*_**. In the remainder of the discussion, we consider several practical issues relating to the implementation of weighted composites in clinical trials.

### 5.1 Missing data and random measurement times

In clinical trial practice, researchers are likely to encounter both variable measurement times and missing observations. In our experience, disagreements between nominal and actual measurement times typical of clinical trials have minimal impact on endpoint efficiency and hence should not generally be of great concern. Missing data, whether due to attrition or to isolated failures in test administration or data collection, is potentially a more significant issue. For example, weights that are optimal for completers are likely to be suboptimal for non‐completers owing to the functional dependence of the LME weights on the design term *τ*_*e*_(***t***). Furthermore, because composite calculation requires complete data at each measurement occasion, the composite test score will be missing if even a single component test score is missing. One option for addressing these concerns is to employ a fully multivariate multiple imputation strategy (i.e., imputation of scores on component tests). In addition to being good practice in the handling of missing data, this option is also well suited to the present setting because it permits calculation of composite scores in the presence of intermittent missing data on individual tests. Other options include calculation of weights using modified formulas that leverage expected or observed dropout patterns rather than the nominal trial design. The relative impacts of these strategies on trial efficiency are as yet unclear, and further studies of composite performance in the presence of missing data will be required.

### 5.2 Composites for between‐group designs

As noted in Section 2, the LME weights given in Equation (2) are optimal for both single‐group and between‐group designs provided the PI assumption is satisfied. Equation (3) is more generally applicable, but less practical because it requires specification of the differential magnitudes of treatment effects across tests. Because of this and in the absence of information to indicate that PI does not hold, we expect that the LME weights of Equation (2) will prove to be of greater utility in most cases. At the same time, the potential for violations of the PI assumption has been cited by some researchers as partial motivation for relying on inverse baseline standard deviation‐weighted composites as an alternative to numerically optimized composites 6. We question whether baseline‐standardized composites offer meaningful protection for this case, however. For example, tests that are subject to ceiling effects may exhibit marked insensitivity to treatment efficacy, yet because they also tend to have restricted variability at baseline, it is precisely these tests that are likely to be most strongly emphasized in inverse baseline standard deviation‐weighted composites.

We describe two ways, one model based and one empirical, in which Equation (3) can be utilized to adjust weights and/or assess efficiency losses tied to PI violations when PI cannot be safely assumed and no data are available to directly inform effect size determination. The first general strategy is to reference covariance parameters for the random slopes in conducting sensitivity analyses for different relative effect sizes on the component tests. For example, we conjecture that when pairwise correlations among random slope coefficients for different tests are high, this may be taken as an indication that the PI assumption is reasonable. This in turn suggests that if the correlations between the random slope coefficients for a given test *A* and those of the remaining tests are all small relative to the intercorrelations among those remaining tests, it may be worth exploring how composite efficiency would be impacted if rate of change on test *A* was unaffected by treatment. Alternatively, sensitivity analyses for composites calculated using Equation (2) could be carried out by assuming that treatment effect sizes are inversely proportional to the ratio of the mean rate of change to the standard deviation of rates of change on each test. There are at least two reasons that such an approach might prove valuable: (1) a pronounced lack of heterogeneity in rate of decline may be taken as an indicator of potential treatment insensitivity, and (2) in so far as LME weights tend to favor tests with a strong signal relative to the amount of between‐subject variability, positing larger effect sizes for tests with weaker signals relative to between‐subject variability should provide a fairly strong test of the robustness of LME‐weighted composites to PI violations.

The second approach we outline is perhaps better suited to weight determination by Equation (3) and builds on the distinction between age‐related and disease‐related decline when normative data are available. To motivate, note that even in the event that an effective treatment for AD is found, it may be that the best‐case scenario for treated patients is a post‐therapeutic rate of decline that is commensurate with that of healthy controls. Accordingly, it may be appropriate to determine weights as in Equation (3), but under a modified form of PI in which ***β***_*Trt*_=*k* ***β***_*CNE*_+(1 − *k*)***β***, where *k*∈[0,1) is a constant and ***β***_*CNE*_ is the mean slope vector for an appropriately matched population of cognitively normal elderly subjects. The proposal in this case is thus to leverage normative data on the component tests to support pre‐specification of effect sizes that respect an invariant proportionality of, on the one hand, the difference between treatment and placebo rates of decline, and, on the other, the difference between healthy control and placebo rates of decline. This approach will tend to downweight tests that are, relative to the other component tests, more sensitive to age‐related declines than to the differences in rate of change between patients and healthy controls.

### 5.3 In‐trial weight estimation

A final issue of some importance concerns the question of whether weights should be estimated and specified in advance of the trial in which they are to be implemented, as we have assumed would be the case. As previously noted, alternative strategies have been suggested or adopted. For example, the primary outcome measure specified for the joint ADCS/Eli Lilly Anti‐amyloid Treatment in Asymptomatic Alzheimer's study 14 is the baseline‐standardized ADCS Preclinical Alzheimer Cognitive Composite, with weights set to be determined from in‐trial baseline data 6. A second more directly relevant example comes from Xiong *et al*. 8, who propose that weights be estimated using data from the trial in which the weights are to be applied and described a procedure in which a multivariate LME model is fit to repeated bootstrap samples to estimate the off‐diagonal blocks of the information matrix of the fixed effects estimates for hypothesis testing purposes. Our preference for pretrial weight estimation of LME weights owes to several factors. First, independently estimated weights can be treated as fixed constants, and hence the resulting composite can be analyzed using methods appropriate for any single prespecified quantitative outcome measure. This means that critical values for hypothesis tests do not need to be adjusted to account for the optimization, and that specialized procedures such as the Xiong *et al*. bootstrap are not required for estimation of standard errors. This latter point is also relevant to the practicability of the trial analysis, as in our experience, failures of convergence in doubly multivariate parameter estimation are relatively common in off‐the‐shelf statistical programs that can be used to fit multivariate LME models. In further support of pretrial weight calculation, the results of Section 4 indicate that minimal efficiency losses are to be expected for composites calculated using weights estimated from independent pilot data given sufficient training sample sizes and provided assumptions are met. There is also some question as to the circumstances under which regulatory agencies will be willing to sanction clinical trials when the construction of the primary endpoint is left unspecified until after the trial has been completed and the data have been unblinded. Finally, we note that in‐trial weight estimation can introduce complications with respect to interpretation and hypothesis testing of the composite slope. Specifically, fully in‐trial estimation of either UTD or LME weights leads to a composite slope *estimate* that is defined as a quadratic form in a positive definite matrix. The slope estimate under this analysis plan is thus inherently nondirectional, with a sampling distribution that is either non‐negative or non‐positive depending on the sign of the normalizing constant *c*, and we conjecture that a failure to account for this in hypothesis testing is likely to have adverse consequences for control over the type I error rate.

## 6 Conclusion

Composite outcome measures can be used to improve statistical power in longitudinal clinical trials. However, these gains can only be realized for linearly weighted composites if the chosen weights are appropriate for the pool of component tests, the study population, and the trial design. This manuscript has presented methods for determining optimal composites under the assumption that the component tests follow a multivariate LME model. The theory that we have outlined can be used not only to determine the optimal weights for a given set of component tests but also to guide selection of component tests for incorporation into the composite. Furthermore, the approach that we have proposed for implementing weighted composites allows the use of standard analytic and hypothesis testing procedures provided that pilot data – such as a previously completed trial in the target patient population – are available, makes use of all available information from the administration of each component test, and can be combined with applications of model‐based rescoring algorithms (e.g., item response theory) to individual tests.

## Supporting information



Supporting info item
