# Supplementary material for: Optimal composite scores for longitudinal clinical trials under the linear mixed effects model
Source: Pharm Stat. 2015 Jul 30;14(5):418–26. doi: 10.1002/pst.1701 (PMC5132034; doi:10.1002/pst.1701)
Supplement: Supplementary file 1 — Supporting info item [file PST-14-418-s001.pdf]

Web Appendix: Optimal Composite Scores for Longitudinal Clinical Trials Under the Linear Mixed Effects Model

M. Colin Ard, Nandini Raghavan and Steven D. Edland

In this Web Appendix we document R code for calculating LME weights from a pilot data set. The supplied code can be directly applied to a simulated data set, *dat*, which is also available for download from the web, and is formatted in standard long form for longitudinal analyses of single outcome variables with columns: id (subject identifier), t (measurement time), and test scores A, Ai, B, and Bi. Data was simulated for $N = 400$ subjects across three annual measurements on two pairs of tests. Parameters for the simulation were taken directly from the analysis of the ADCS MCI/Donepezil trial[9] presented in Section 3 of the manuscript, with test A corresponding to the ADAS, and test B corresponding to the CDR. Tests Ai and Bi were calculated using the same random effects used in the calculation of tests A and B, respectively, but with residual errors simulated under the assumption of complete independence. The true LME weights for the chosen parameter set and trial design, normalized to sum to 1, are $\boldsymbol{w}_{LME}([0,1,2]^T) = [0.681, 0.319]$ for tests A and B, and $\boldsymbol{w}_{LME}([0,1,2]^T) = [0.684, 0.316]$ for tests Ai and Bi.

When pilot data from a study incorporating the same design as the planned trial is available, balanced and complete (or can be made so by imputation), a straightforward procedure is to estimate the parameters using a *summary measures* approach in which $\hat{\boldsymbol{\beta}} = N_{Pilot}^{-1} \sum \hat{\boldsymbol{\beta}}_i$, and $\hat{\boldsymbol{\Lambda}}_{\boldsymbol{t}} = N_{Pilot}^{-1} \sum (\hat{\boldsymbol{\beta}}_i - \hat{\boldsymbol{\beta}})(\hat{\boldsymbol{\beta}}_i - \hat{\boldsymbol{\beta}})^T$, with $\hat{\boldsymbol{\beta}}_i$ the ordinary least squares slope estimate based on data from the ith subject, and N_{Pilot} the sample size for the pilot study. As implied, $\hat{\boldsymbol{\Lambda}}_{\boldsymbol{t}}$ is the estimated covariance matrix of the $\hat{\boldsymbol{\beta}}_i$ (*cf.*, Appendix A). Note that in the event that change on the composite from baseline to time T is to be specified as the trial endpoint it may be necessary to subset the pilot data to include only the baseline and time T measurements before calculating the $\hat{\beta}_i$'s. The required calculations can be carried out as follows using the R package *nlme*[16]:

```
fit<-cbind(coef(lmList(A~t|id,data=dat))$t,
      coef(lmList(B~t|id,data=dat))$t)
w<-c(solve((dim(dat)[1]-1)*cov(fit)/dim(dat)[1])%*%colMeans(fit))
w<-w/sum(w)
```

Applying this code to *dat* yields $\hat{\boldsymbol{w}}_{LME}([0,1,2]^T) = [0.672, 0.328]$. Substituting in tests Ai and Bi in place of A and B, the estimated weights are $\hat{\boldsymbol{w}}_{LME}([0,1,2]^T) = [0.626, 0.374]$.

Another option for estimating parameters is to use the R function `lme()`, again from the *nlme* package[16]. Unlike the summary measures approach outlined above, this procedure directly estimates the variance components of the $\boldsymbol{\Lambda}_{\boldsymbol{t}}$ matrix, which can then be used to determine weights for any desired trial design for which the model is assumed to hold. To use this approach the data should be placed in a stacked long form with

columns `id`, `t`, `test` (identifying the component test associated with each row), and `y` (a numeric variable giving the observed score). Code for reformatting is follows:

```
tempAi<-subset(dat,,c(id,t,Ai))
     names(tempAi)[3]='y'
     tempAi$test='Ai'
tempBi<-subset(dat,,c(id,t,Bi))
     names(tempBi)[3]='y'
     tempBi$test='Bi'
long<-rbind(tempAi,tempBi)
```

With data in this form, code for fitting a model for two tests under the assumption of independent residual errors, extracting parameters, and calculating weights is as follows:

```
fit<-lme(y~ 0 + I(as.numeric(test%in%'Ai')) +
          I(as.numeric(test%in%'Bi')) + I(as.numeric(test%in%'Ai')*t) +
          I(as.numeric(test%in%'Bi')*t),
     data = long,
     random = ~ 0 + I(as.numeric(test%in%'Ai')) +
          I(as.numeric(test%in%'Bi')) + I(as.numeric(test%in%'Ai')*t) +
          I(as.numeric(test%in%'Bi')*t) | id,
     weights = varIdent(form = ~ 1 | test),
     control=lmeControl(opt='optim') )
M.slpest<-fixef(fit)[3:4]
s.slpest<-diag(VarCorr(fit)[3:4,'StdDev'])
Cor.slpest<-matrix(c(1,rep(as.numeric(VarCorr(fit)[4,5]),2),1), nrow=2)
S.slpest<-s.slpest%*%Cor.slpest%*%s.slpest
S.eest<-diag( as.numeric(VarCorr(fit)['Residual','Variance'])*
     c(1,coef(fit$modelStruct$varStruct,uncons=F)[[1]]^2) )
w<-solve(S.slpest+tau*S.eest)%*%M.slpest
w<-w/sum(w)
```

In the code above we have used the `control` argument of `lme()` to achieve convergence. Applying the code above to `dat` yields weight estimates $\hat{\boldsymbol{w}}_{LME}([0,1,2]^T) = [0.624, 0.376]$.

The final option we consider for estimating LME weights uses the `growth()` function from the R structural equation modeling package *lavaan*[11]. This analysis requires that data be put in wide form with one row per subject and one column for each test at each measurement occasion, as follows:

```
wide<-data.frame(id=unique(dat$id))
for(j in unique(dat$t))
     {
     foo<-subset(dat,t==j,c(id,A,B))
     names(foo)[2:3]<-paste(c('A','B'),j,sep='.')
     wide<-merge(wide,foo)
     }
```

With data in the appropriate form and labeled as above, parameters can be estimated and extracted, and weights calculated as follows:

```
mdl<-'iA =~ 1*A.0 + 1*A.1 + 1*A.2
     sA =~ 1*A.1 + 2*A.2
     iB =~ 1*B.0 + 1*B.1 + 1*B.2
     sB =~ 1*B.1 + 2*B.2
     A.0 ~~ vA*A.0 \n A.1 ~~ vA*A.1 \n A.2 ~~ vA*A.2
     B.0 ~~ vB*B.0 \n B.1 ~~ vB*B.1 \n B.2 ~~ vB*B.2
     A.0 ~~ c*B.0 \n A.1 ~~ c*B.1 \n A.2 ~~ c*B.2
     A.0 + A.1 + A.2 + B.0 + B.1 + B.2 ~ 0*1'
fit<-growth(mdl, wide)
M.slpest<-coef(fit)[c('sA~1','sB~1')]
S.slpest<-matrix(coef(fit)[c('sA~~sA','sA~~sB','sA~~sB','sB~~sB')],
     nrow=2)
S.eest<-matrix(coef(fit)[c('vA','c','c','vB')],nrow=2)
w<-solve(S.slpest+tau*S.eest)%*%M.slpest
w<-w/sum(w)
```

In the code above, the object `mdl` specifies the assumed model structure, which in this case includes correlated residuals between tests and within measurement occasions. To specifiy a model with independent residuals, as for an analysis of Ai and Bi, one need only delete the second to last line of this model specification. Applying the supplied code to *dat* yields the weight estimates $\hat{\boldsymbol{w}}_{LME}([0,1,2]^T) = [0.672, 0.328]$.
